# Rhythmic Fluctuations in Tactile Attention

**DOI:** 10.1111/ejn.70247

**Published:** 2025-09-14

**Authors:** Burcu Bayram, Ulrich Ansorge, Ulrich Pomper

**Affiliations:** ^1^ Department of Cognition, Emotion, and Methods in Psychology, Faculty of Psychology University of Vienna Vienna Austria; ^2^ Vienna Cognitive Science Research Hub University of Vienna Vienna Austria; ^3^ Research Platform Mediatised Lifeworlds University of Vienna Vienna Austria

**Keywords:** alpha oscillations, rhythmic processing, spatial attention, tactile

## Abstract

There is increasing evidence that sensory information in the visual and auditory domains is processed not in a continuous but in a rather rhythmically fluctuating fashion, reflecting underlying oscillations in neural excitability. So far, little is known about whether a similar mechanism is also implemented in the somatosensory modality, which, given its importance for monitoring and protecting our bodily integrity, might sample sensory input differently. Here, we investigated (1) whether tactile detection performance fluctuates over time at the speed of somatosensory alpha‐ and beta‐band oscillations and (2) whether attention samples two simultaneously monitored body parts at different time points, similar to previous demonstrations in the visual and auditory domains. Thirty‐one human participants (female and male) performed a behavioral dense‐sampling experiment, consisting of a tactile cuing task, in which they responded to tactile vibrations at detection threshold presented to the left or right index fingers. In line with our hypotheses, we observed temporal fluctuations in both hit rates and response times, at a speed of 8–16 Hz, consistent with electrophysiological alpha‐ and beta‐band oscillations commonly observed over somatosensory areas. Further, hit rate fluctuations exhibited phase differences at ~13 Hz between the left and right hands, suggesting that somatosensory spatial attention samples cued and uncued locations at different points in time. Overall, along with recent work, which identified markers of attentional sampling also beyond sensory processing, our study provides novel evidence that cyclical information processing and alternating sampling of simultaneously attended entities might be a general mechanism in the brain.

## Graphical abstract

Rhythmic fluctuations in attention are well established in the human visual and auditory domains. Here, we demonstrate a similar mechanism in the tactile modality. Detection accuracy and response times towards tactile stimuli fluctuated at the speed of alpha and beta oscillations, prominently linked to tactile attention. Further, accuracy at two simultaneously monitored locations fluctuated at different phase angles, suggesting that tactile attention cyclically alternates between locations.

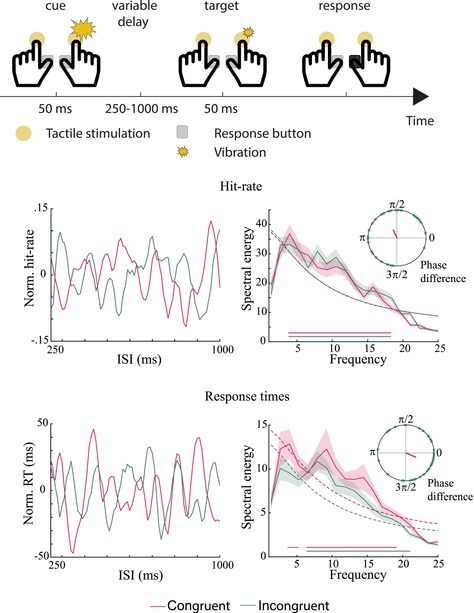

**Abbreviations**
Con: congruent
EEG: electroencephalography
FA: false alarm
FDR: false discovery rate
FFT: fast Fourier transform
Hz: Hertz
Inc: incongruent
ISI: inter‐stimulus interval
M: mean
MEG: magnetoencephalography
ms: millisecond
RT: response time
SD: standard deviation
Thresh.: threshold

## 1 Introduction

Although we subjectively perceive the world around us in a continuous fashion, recent evidence suggests that our brain samples incoming sensory information in a more discrete, step‐by‐step manner, similar to individual frames in a movie (Fiebelkorn and Kastner 2019; Landau 2018; VanRullen 2016). Research assumes that rhythmic oscillations of neural ensembles—reflecting cyclical modulations of neural excitability and thereby creating more or less favorable moments for information processing—underlie these perceptual snapshots (Busch et al. 2009; Samaha and Postle 2015). Behavioral studies have demonstrated these rhythmic fluctuations in performance at around 4–12 Hz in the visual and auditory domains, using the so‐called “dense sampling” paradigms (e.g., Fiebelkorn and Kastner 2019; Landau 2018; Pomper and Ansorge 2021, 2023; Re et al. 2019; VanRullen 2016). Here, the phase of ongoing attentional fluctuations is reset via a salient cue at the beginning of each trial. Following a delay interval, which varies in small steps across trials, a target stimulus is presented. Finally, analyzing performance as a function of the cue‐to‐target interval can reveal potential rhythmic fluctuations in performance.

Recent studies have used such paradigms to show that when humans monitor several potentially relevant locations or objects simultaneously, attention not only fluctuates rhythmically, but seems to switch back and forth between locations in alternation, at a rate of between 4 and 12 Hz (Fiebelkorn et al. 2013; Helfrich et al. 2018; Landau and Fries 2012). In an exemplary study, Landau and Fries (2012) presented their participants with an exogenous spatial cue to either a left or right potential visual target location. By densely varying intervals between cue and target across trials, the authors were able to reveal rhythmic fluctuations in detection performance between 4 and 10 Hz at each location. Moreover, the performance fluctuations at the two locations were in anti‐phase, suggesting that attention samples them in alternation. Using a similar approach, we have recently demonstrated analogous fluctuations between two items in visual working memory, suggesting that cyclic sampling of information might be one of the brain's key mechanisms in stimulus or information selection, against the backdrop of limited information processing resources or other functional constraints (Pomper and Ansorge 2021; Schmid et al. 2022).

So far, attentional sampling has almost exclusively been demonstrated within the visual and auditory modalities (but see Baumgarten et al. 2015, 2017, discussed below). At the same time, it is well established that rhythmic neural activity also plays a crucial role in the processing of somatosensory information. Specifically, many electrophysiological studies have reported oscillations in the alpha (8–14 Hz) and lower beta ranges (15–25 Hz) as a correlate of somatosensory attention, with activity in these bands decreasing following stimulation, but also already in anticipation of a task‐relevant stimulus (Haegens et al. 2011; Jasper and Andrews 1936; Jones et al. 2010; Keil et al. 2016; Pomper et al. 2013, 2015). Thus, research suggests that similar to the visual and other sensory modalities, alpha‐ (and beta‐) band activity plays a critical role in sensory gating in the somatosensory system (Neuper et al. 2006). Consequently, it seems probable that somatosensory attention and subsequent detection performance similarly fluctuate over time, presumably at the speed of the prominent neural alpha and beta oscillations.

Evidence for such rhythmic sampling in the somatosensory domain comes from Baumgarten et al. (2015), who presented participants with two electrodermal impulses placed 25 ms apart. Whether they perceived the stimuli as one single or two separate events depended on the phase of neural oscillations in the alpha‐band and low beta‐band (8–20 Hz) in somatosensory cortex prior to the stimulation, as measured via magnetoencephalography. This suggests a limited temporal resolution for tactile information resulting from discrete perceptual time windows, with two tactile stimuli being perceived as one if they fall within a single perceptual cycle. In a subsequent study, Baumgarten et al. (2017) further demonstrated that following a subliminal tactile cue, the likelihood of perceiving two subsequent tactile stimuli as one fluctuated rhythmically at the speed of beta‐band oscillations.

Although these studies provided promising support for a cyclic sampling in the somatosensory domain, it currently remains unclear (1) whether ongoing somatosensory detection performance also fluctuates rhythmically, and (2) whether several simultaneously relevant somatosensory locations are sampled in alternation (analogously to visual and auditory locations or contents in working memory; Fiebelkorn et al. 2013; Ho et al. 2017; Landau and Fries 2012; Pomper and Ansorge 2021).

In the present experiment, we addressed this gap in current knowledge as well as sought to corroborate related earlier findings in the somatosensory domain by combining a tactile cuing paradigm with a behavioral dense sampling approach. On each trial, participants received an uninformative tactile cue to the left or right index finger, followed first by a variable delay (250 to 1000 ms) and then a target either spatially congruent or incongruent with the cue. We observed rhythmic performance fluctuations in both hit rate and reaction times (RTs) in the range of 8 to 16 Hz, as well as different phase angles of hit rates at ~13 Hz between spatially congruent and incongruent targets. Our results indicate that somatosensory attention indeed works in a cyclic fashion, with multiple spatial locations being sampled at different points in time.

## 2 Materials and Methods

### 2.1 Participants

Thirty‐two participants took part in the study either in exchange for course credits or monetary compensation. One participant did not return for the second data collection session and was therefore excluded from further analyses. The sample size was based on previous attentional‐sampling studies, many of which have included between 20 and 30 participants (e.g., Pomper and Ansorge 2021; Re et al. 2019). Although we did not explicitly consider effect sizes in determining our sampling size, we expected a medium‐sized effect based on the results from one of our previous studies (Pomper and Ansorge 2021), in which the presumed fluctuations in working‐memory guided visual detection performance had Cohen's D values of 0.39, 0.36, and 0.07. In case of a regular, one‐tailed paired *t*‐test with an alpha of 0.05 and a power of 0.8, the required sample size to detect an effect of Cohen's D = 0.37 and 0.39 would be 36 and 33, respectively, and 1178 for an effect of Cohen's D = 0.07 (via G*Power, Faul et al. 2007; unfortunately, testing the required sample size for the latter finding is beyond our possibilities). However, in our present case, we are comparing empirical data with the mean of 1000 permutations of those empirical data. As a consequence, the variance of the permutation mean will be much lower than that of the empirical mean, leading to a smaller variance in the condition difference scores and thus a larger test power. In other words, the required sample size for such a *t*‐test of empirical against permutation data will need a smaller sample size to detect a true effect compared with a regular t‐test. To our knowledge, there is no readily available toolbox or formula that implements such a calculation, but we would like to argue that our current sample size of *N* = 31, which is close to the required size for a regular t‐test, will suffice given the increased power of our specific calculation.

The critical effect size for our current study, again computed via G*Power and assuming an alpha of 0.05 and a power of 0.8, is *d* ≈ 0.42. However, following our argument above, this value is likely to be lower in our special case of testing against the mean of permutation data.

The remaining 31 participants (11 males, *M*_age_ = 22.2, SD_age_ = 2.5) were naïve to the purpose of the experiment. All gave written informed consent, and the study was conducted in accordance with the standards of the Declaration of Helsinki. We further followed the Austrian Universities Act of 2002, which states that only medical universities or studies conducting applied medical research are required to obtain additional approval by an ethics committee. Therefore, no additional ethical approval was required for our study.

### 2.2 Experimental Setup and Task

We ran the experiment using OpenSesame (version 3.2.8; Mathôt et al. 2012) on a Windows 7 computer. Participants sat at a desk in a dimly lit room in front of a computer screen, with their head supported by a chin‐ and headrest. The tactile stimuli were delivered via a “tactor” vibrational unit (Dancer Design, UK) controlled via the computer's sound card. The stimulus timing was accurate in the range of ± 3 ms, as verified via an oscilloscope connected to the computer's sound output. Participants placed their left and right thumbs on the outer “ctrl” and “enter” keys, respectively, of a regular “QWERT” keyboard. Each index finger was placed on top of a tactor unit located left and right next to the keyboard, resulting in a distance of approximately 50 cm between the index fingers. To mask potential acoustic noise from the tactile stimulator, participants wore earmuffs. Additionally, we placed the tactor units on cotton pads for further reduction of any possible vibrational noise. The authors extensively piloted the experiment before the data collection and did not perceive any noise from the vibrational unit. Upon completion of the experiment, we asked each participant whether they had heard any sounds from the stimulation, which none of them reported. Thus, we are confident that noise from the tactile stimulator was inaudible and not an issue in our present study.

**FIGURE 1:**
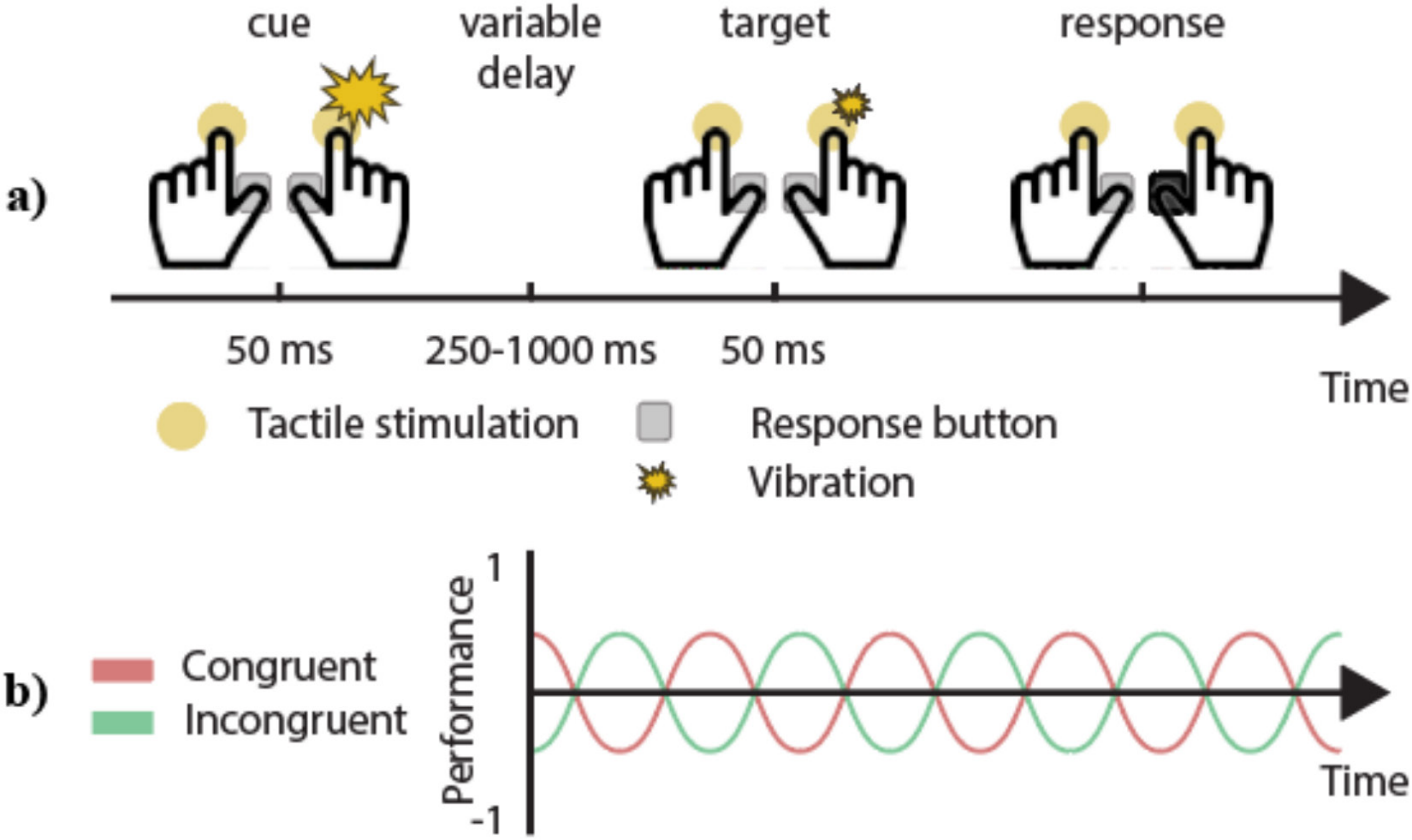
(a) Experimental design: At the beginning of each trial, participants received an uninformative tactile cue to the left or right index finger. After a variable delay, a tactile target close to detection threshold was presented to either the cued or to the opposite hand. The task was to indicate the side of the target stimulus as fast and accurately as possible via a button press. (b) Hypothesized results: We expected that target detection performance would cyclically fluctuate over time following the cue and that performance for congruent and incongruent trials would be out‐of‐phase.

Throughout the experiment, participants fixated on a black dot at the center of a gray screen. At the start of each trial (see Figure 1a), an uninformative tactile cue (50 ms above‐threshold vibration) was delivered to the left or right index finger (each 50% probability). We used noninformative spatial cues because the investigated attentional fluctuations are commonly small in amplitude (Fiebelkorn et al. 2013; Ho et al. 2017; Landau and Fries 2012; Pomper and Ansorge 2021) and thus arguably most effective and easiest to detect when stimuli are presented close to detection threshold. Moreover, the presumed attentional fluctuations can affect unconscious processing and subsequent behavior, even on trials in which participants do not have a conscious perception of the target. Past studies have effectively used noninformative spatial cues to attract initial exogenous spatial attention (Lou et al. 2022) and to study the subsequent alternate sampling of two simultaneously monitored locations (Landau and Fries 2012; Michel et al. 2021; Plöchl et al. 2022). Specifically, rather than changing the overall focus of spatial attention throughout a trial, the cue in dense‐sampling experiments is intended to reset spatial attention to one side at a known time point at the beginning of each trial, allowing for the testing of the presence of the hypothesized subsequent cyclic shifts of attention between the locations. To this end, the rationale behind using uninformative cues is to have a more equal balance of responses to the cued and uncued side.

Next, we presented a variable delay of 250–1000 ms varied in 10‐ms steps, thus resulting in 76 potential delay intervals. Crucially, testing detection performance at many different delay intervals following the spatial cue allowed us to estimate the time course of somatosensory attention. Finally, a tactile target (50‐ms vibration close to detection threshold) was delivered either to the cued hand (congruent trials) or the opposite hand (incongruent trials; each 50% probability). Participants reported a perceived target by pressing the keyboard button via the thumb of the respective hand as accurately and fast as possible. We adaptively adjusted target stimulus intensity every nine trials to yield a performance significantly above but close to 50% (within target‐present trials) in order to keep the task demanding and maintain performance variability (Fiebelkorn et al. 2013; Helfrich et al. 2018; Pomper and Ansorge 2021). Specifically, target stimulus volume was automatically increased (decreased) by 0.5% of maximum sound volume in OpenSesame if less (more) than five of the nine previous target‐present trials were correct. After each trial, participants received visual feedback on whether their response was correct or incorrect. Overall, we presented 10 trials per condition (congruent, incongruent) for each of the 76 variable delay intervals. This number of trials has been shown to be sufficient for the detection of behavioral oscillations (Helfrich et al. 2018; Pomper and Ansorge 2021; Schmid et al. 2022). Additionally, we presented 280 trials containing no target to estimate the participants' false‐alarm rate. We explicitly informed participants about these trials and instructed them to only press a button when they were certain that a target had appeared. The order of trials was randomized across participants. Self‐timed breaks were administered every 60 trials, during which feedback on the average accuracy and RT in the previous block was presented. In total, the experiment comprised 1800 trials, collected in two sessions on separate days.

Prior to the experiment, we assessed individual thresholds for the perception of the tactile target stimuli through an up‐and‐down staircase procedure via 50 trials and used the value of the final trial. On each trial, participants were presented with a single tactile stimulus to either the left or right hand (50% each) and, as in the main experiment, had to indicate which hand was stimulated via a button press. Stimulus intensity started out at a clearly perceivable level (10% of the cue level in the main experiment) and was reduced following each correct trial and increased following each incorrect trial (in steps of 2.5% of the initial volume). Additionally, participants completed 20 practice trials to familiarize themselves with the task.

### 2.3 Data Analysis

We performed all data analyses using MATLAB (version 2018; Mathworks, Natick, MA, USA) and the CircStat toolbox (version 1.21; Berens 2009). First, we removed trials in which participants accidentally responded prior to the target (e.g., due to responding to the cue instead of the target; *M* = 14.2 trials; SD = 31.9). As a basic descriptive analysis, we computed mean hit rates and RTs (only for correct responses) for all trials pooled together, as well as separately for the congruent and incongruent conditions, and compared performance between the conditions via paired‐samples *t*‐tests.

Next, we investigated the time course of hit rates and RTs as a function of the cue‐to‐target delay interval, again for all trials pooled together as well as separately for the congruent and incongruent conditions. To do so, we first sorted the trials for each participant according to their delay interval duration. Then, we used a moving‐window averaging approach (step size of 10 ms) and computed the mean hit rates and RTs within bins of five consecutive delay‐period intervals (i.e., 50 ms in total per bin; see, e.g., Fiebelkorn et al. 2013; Helfrich et al. 2018; Plöchl et al. 2022; Pomper and Ansorge 2021), separately for the pooled data, the congruent, and the incongruent conditions. Subsequently, we detrended and normalized each single subject's time course of hit rates and RTs by subtracting the second order polynomial fit and performed fast Fourier transform (FFT) to estimate their spectral composition. For each subject, this yielded both power and phase values for 19 frequency bins from 1.3 to 25 Hz. We did not apply a windowing function prior to the FFT because (1) our data segments are already very short (76 samples) and tapering would remove further information at the edges, and (2) we see genuine energy/fluctuations at both ends of the time series (see Figure 3), which would be shaved off by a taper. However, we reanalyzed our data tapered by a Kaiser window (with a beta parameter of 1), which is not as steep as a Hanning or Bartlett window but still reduces edge artifacts and spectral leakage compared with using no taper. This analysis yielded the same overall results as our present analysis, with all significant effects retained and only differing in the specific numerical outcomes.

To assess the statistical significance of spectral power in the performance time courses of hit rates and RTs, we applied a nonparametric resampling procedure (Fiebelkorn et al. 2013; Landau and Fries 2012; Pomper and Ansorge 2021). Specifically, we created 1000 surrogate datasets under the null hypothesis that no periodic temporal pattern is present in the performance time series, by fitting each participant's time series with an auto‐regressive model with a single coefficient (Brookshire 2022; Harris and Beale 2024):

$$X_{t}=\delta +\varphi_{1}X_{t-1}+\varepsilon_{t}$$

Here, 𝑋_𝑡_ denotes the value of the time series at time *t*, 𝛿 is a constant, 𝜑_1_ is the autoregressive coefficient of the model that describes how the data at time *t* − 1 influence time t, and 𝜀_𝑡_ is a noise term. We then used this model to generate 1000 surrogate time‐courses for each condition and participant, with similar aperiodic properties to the original data, which we likewise transformed to the spectral domain via FFT (Harris and Beale 2024).

Finally, for each condition and frequency band, we compared the empirical spectral power with the mean of the surrogate spectrum via *t*‐tests (*p* = 0.05), and applied false‐discovery rate (FDR) correction to compensate for multiple comparisons across frequency bands (Benjamini and Hochberg 1995).

To test our second hypothesis, we were interested in potential phase differences between performance fluctuations for the congruent versus incongruent condition, as an indication of an alternating distribution of attention. To this end, for each significant frequency in the power spectrum, we computed the circular distance between the congruent and incongruent condition's phase angle on a single‐subject level. Then, we tested whether the resulting differences were distributed non‐uniformly around the unit circle across participants using Rayleigh's test for non‐uniformity (Berens 2009). Notably, in contrast to comparing the phase angles between the individual conditions via, for example, a Watson–Williams test (Berens 2009), this approach can reveal consistent phase relationships between conditions even when the individual congruent and incongruent conditions do not exhibit phase congruence across participants. In other words, our approach is sensitive to cases in which the phase difference between the performance fluctuations in the congruent and incongruent conditions is consistent across participants. Finally, to test whether phase differences are not only consistent across participants but also different from zero, we computed a *v*‐test against zero for both hit rates and response times. Similar to the spectral power analyses, these phase consistency tests were conducted both on the empirical and the surrogate datasets, and statistical significance was assumed if the *p*‐value from the empirical data exceeded 95% of *p*‐values in the surrogate data.

## 3 Results

**FIGURE 2:**
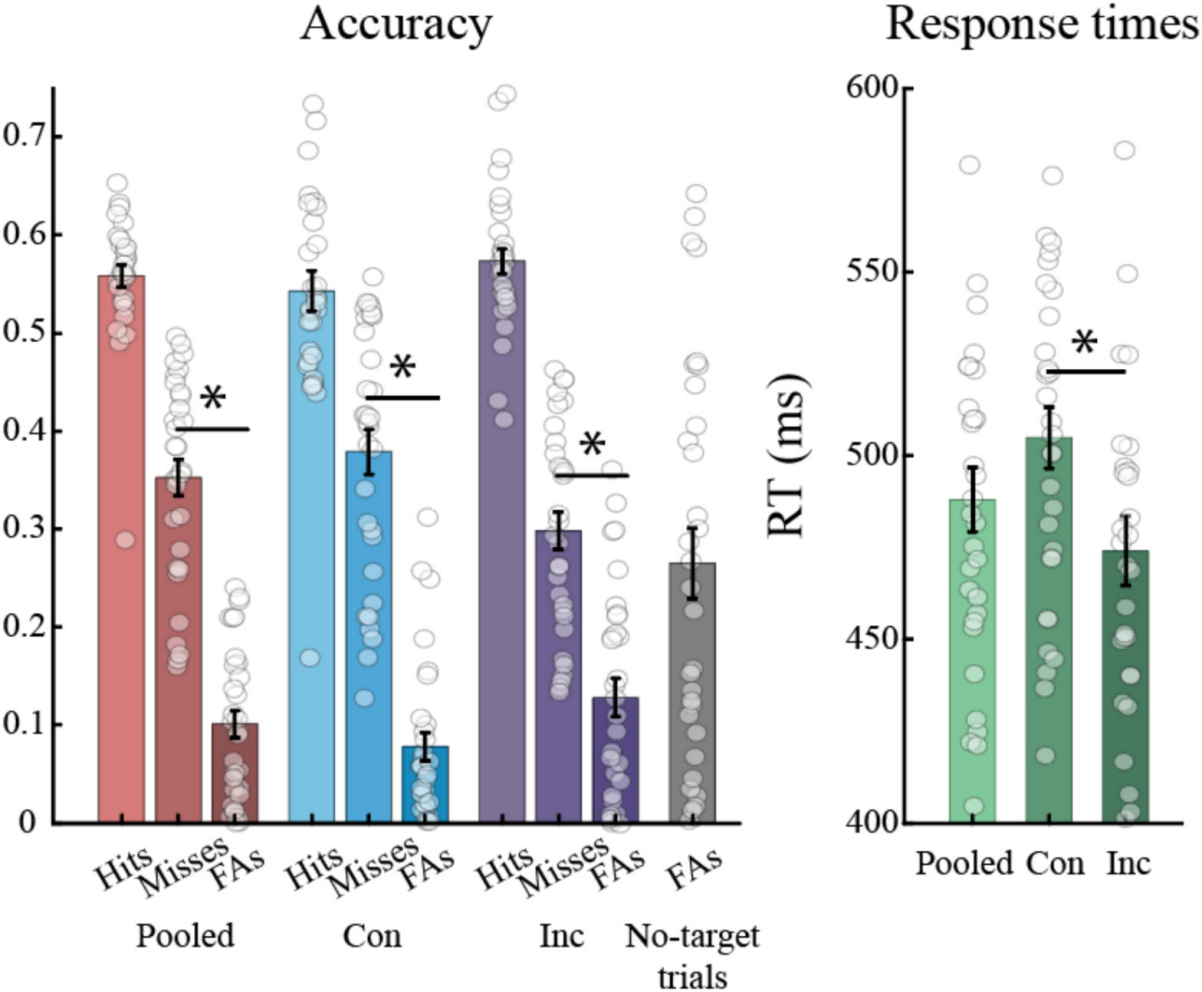
Left: performance accuracy for all trials pooled together (pooled), as well as for the congruent (Con) and incongruent (Inc) conditions and the no‐target trials. Separate bars show hit rates, misses, and false alarms (FAs). Right: response times for all trials pooled together, as well as for the congruent and incongruent conditions. Error bars indicate the SE of the mean, circles represent individual participants. Asterisks indicate significant differences.

Participants achieved an average hit rate of 55% (SD = 6%) and an RT of 488 ms (SD = 49 ms) (Figure 2). We observed significantly faster RTs for incongruent trials (*M* = 474 ms, SD = 53 ms) compared with congruent trials (*M* = 505 ms, SD = 47 ms), *t*(30) = 6.93, *p* < 0.001, but no significant difference in hit rates between incongruent (*M* = 56.9%, SD = 8%) and congruent trials (*M* = 54%, SD = 11%; *p* = 0.26).

Further, our data show that, although hit‐rate was only slightly above 50%, most errors were due to withheld responses (misses) rather than incorrect responses (false alarms). The difference between misses and false alarms was significant for the pooled data, *t*(30) = 8.35, *p* < 0.001, as well as for the separate congruent and incongruent conditions, *t*(30) = 9.12, *p* < 0.001 and *t*(30) = 4.65, *p* < 0.001, respectively. This indicates that our participants were not guessing, but adhered to the instructions of responding only when they were certain that a target had appeared.

**FIGURE 3:**
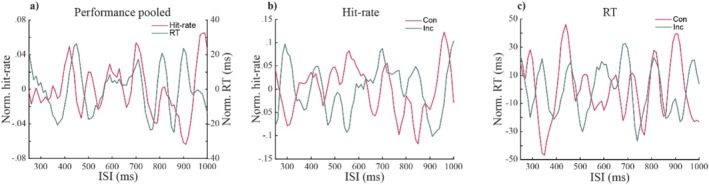
Performance time‐courses from one exemplary subject (smoothed; see Figure S1 for the unsmoothed and undetrended data and Figure S2 for the remaining subjects). (a) The performance time courses show hit‐rates (pink trace) and response times (RTs; green trace) as a function of the variable inter‐stimulus interval (ISI), pooled across all trials. (b) The hit‐rate time course for the congruent (pink trace) and incongruent (green trace) conditions as a function of the variable inter‐stimulus interval (ISI). (c) Same as Part (b), for RTs.

**FIGURE 4:**
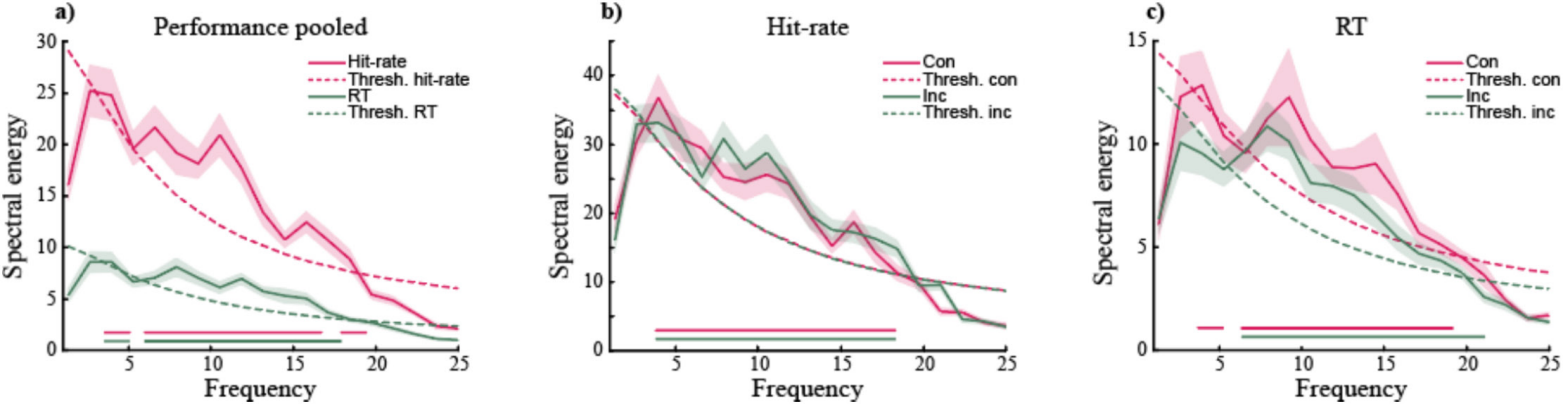
(a) The grand‐average spectral representation of performance fluctuations pooled across all trials, with pink traces representing hit‐rate and green traces representing response times (RTs). Empirical data is presented in solid lines, the significance threshold (thresh.) determined from resampled data in dashed lines. (b) The grand‐average spectral energy of the time course of hit‐rates, with pink traces representing congruent and green traces representing incongruent trials. Empirical data is presented in solid lines, the significance threshold (thresh.) determined from resampled data in dashed lines. (c) Same as (b), for response times (RTs). Shaded areas indicate standard error of the mean. Colored horizontal lines indicate frequency bins with significant spectral power.

In the individual participant's performance time courses (Figures 3 and S1), we found that both hit rates and RTs fluctuate across time for data pooled across all trials, as well as for the congruent and incongruent conditions separately. Importantly, in line with our first hypothesis, these fluctuations exceeded chance level, as shown via our spectral analysis (Figure 4; Table 1). Pooled across all trials, we observed significant spectral power for hit rates between 6.6 and 17.1 Hz and for RTs between 7.9 and 15.8 Hz (Figure 4a). Furthermore, hit rate data showed significant spectral power between 6.6 and 15.8 Hz for congruent trials and between 7.9 and 18.4 Hz for incongruent trials (Figure 4b). RT data showed significant spectral power between 9.2 and 15.8 Hz for congruent trials and between 7.9 and 15.8 Hz for incongruent trials (Figure 4c).

**TABLE 1:** Statistical results (*p*‐values) from the spectral (top) and phase analysis (bottom) for all conditions (rows) and frequencies (columns).

Frequency	6.6 Hz	7.9 Hz	9.2 Hz	10.5 Hz	11.8 Hz	13.2 Hz	14.5 Hz	15.8 Hz	17.1 Hz	18.4 Hz
Spectral power										
Hit‐rate joint	0.035*	0.048*	0.007*	0.001*	0.001*	0.030*	0.120	0.005*	0.035*	0.172
Hit‐rate congruent	0.026*	0.049*	0.049*	0.005*	0.002*	0.011*	0.091	0.003*	0.088	0.680
Hit‐rate incongruent	0.342	0.001*	0.009*	0.001*	0.001*	0.013*	0.013*	0.009*	0.009*	0.006*
RTs joint	0.343	0.014*	0.008*	0.014*	0.001*	0.001*	0.014*	0.003*	0.165	0.760
RTs congruent	0.930	0.104	0.034*	0.034*	0.008*	0.008*	0.017*	0.017*	0.137	0.350
RTs incongruent	0.058	0.004*	0.001*	0.031*	0.004*	0.006*	0.004*	0.031*	0.161	0.124
Phase consistency										
Hit‐rate con vs. Inc	—	0.309	0.623	0.746	0.574	0.028*	—	0.279	—	—
RTs con vs. Inc	—	—	0.279	0.604	0.008*	0.428	0.996	0.808	—	—

*Note:* Only frequencies containing at least one significant result are shown. Asterisks indicate significant data points (FDR corrected).

**FIGURE 5:**
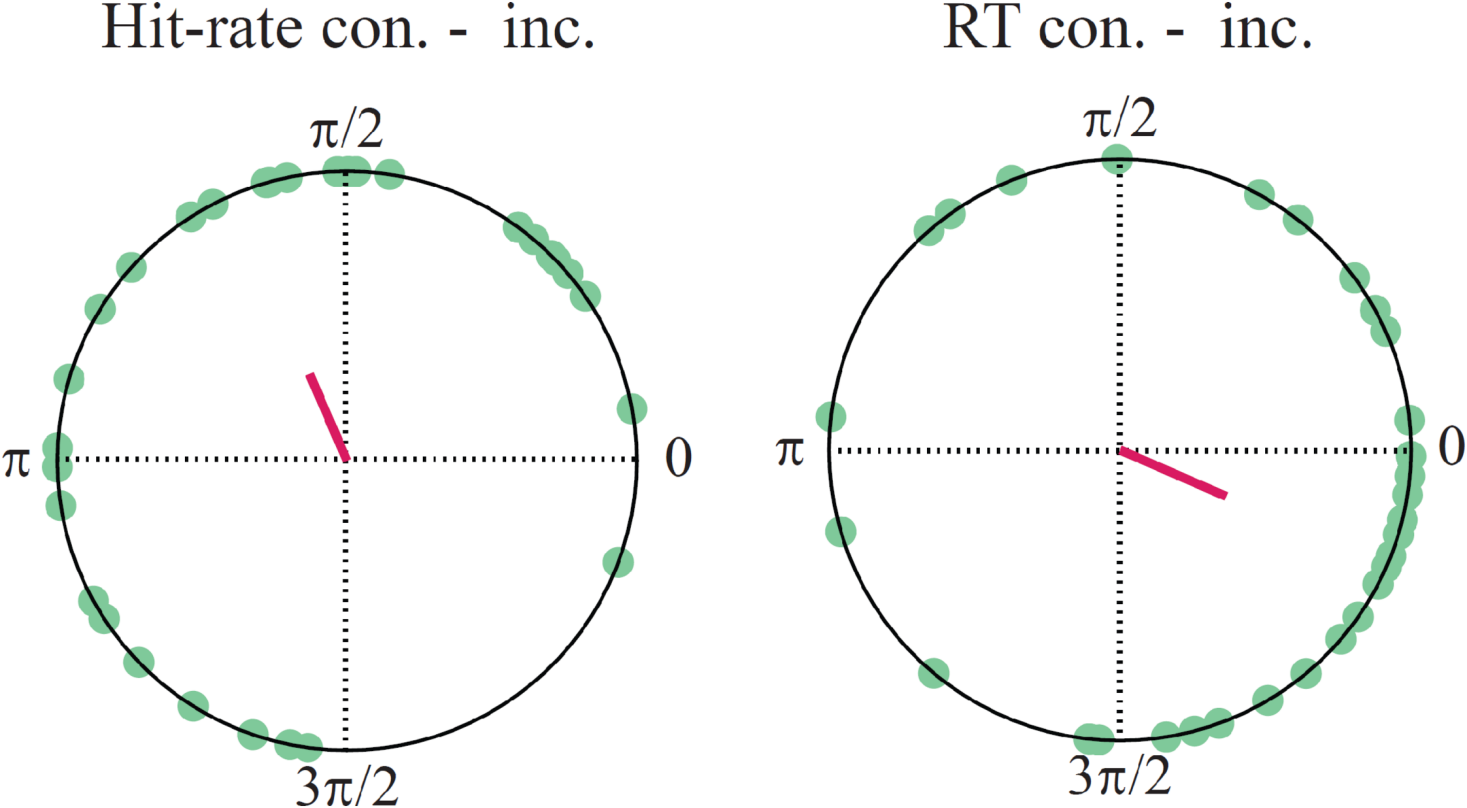
Circular difference between phase angles of the congruent and incongruent conditions, for hit‐rate at 13.2 Hz (left) and response times at 11.8 Hz (right). Green dots indicate individual participant's angles, pink lines represent mean angles.

Finally, for frequencies at which the spectral power was significant for both the congruent and incongruent conditions, we tested whether the circular difference in phase angles between conditions was similar across participants and different form zero (Figure 5; Table 1). The Rayleigh test indicated significant phase consistency of the condition differences for both hit‐rates at 13.2 Hz and RTs at 11.8 Hz. Further, in partial support of our second hypothesis, *v*‐tests showed that the RT phase differences clustered significantly around zero (*p* < 0.02), whereas the hit‐rate phase differences did not (*p* < 0.69). Together with the significant Rayleigh test results, this indicates that hit‐rate, but not RT phase differences are significantly clustered in a direction other than zero, with an average phase difference of ~100° (Figure 5).

Notably, these phase effects are not confounded by power differences between the congruent and incongruent conditions at each relevant frequency (*t*[30] = −0.022, *p* = 0.91 and *t*[30] = 0.837, *p* = 0.42, respectively).

## 4 Discussion

In the present study, we investigated rhythmic information processing in the somatosensory domain using a behavioral dense sampling paradigm. In line with our first hypothesis, we found cyclic performance fluctuations in the alpha and low beta frequency range (~8–16 Hz), supporting a rhythmic attentional sampling mechanism. Further, consistent with our second hypothesis, we observed a non‐zero phase difference between the hit‐rate time course in congruent and incongruent conditions at 13 Hz, indicating that somatosensory attention sampled the two simultaneously monitored spatial locations at different points in time.

### 4.1 Somatosensory Performance Fluctuates at an Alpha/Beta Rhythm

As our first main result, we observed rhythmic temporal fluctuations in hit rates and RTs towards near‐threshold somatosensory stimuli, following an uninformative spatial cue.

This finding aligns with previous studies demonstrating rhythmic fluctuations in visual and auditory attention (Fiebelkorn et al. 2013; Helfrich et al. 2018; Ho et al. 2017; Landau and Fries 2012). However, while the reported oscillations in the visual and auditory domains were commonly at a speed of 4–12 Hz, our present somatosensory paradigm yielded slightly faster fluctuations in the range of neural alpha and low beta frequencies. Crucially, this speed matches with the neural activity predominantly modulated over somatosensory areas during respective tactile attention tasks (Haegens et al. 2011; Jasper and Andrews 1936; Jones et al. 2010; Keil et al. 2016; Pomper et al. 2013, 2015). For instance, Pomper et al. (2013) observed significantly reduced electroencephalography (EEG) beta‐band power in early somatosensory areas when participants directed their anticipatory attention to an upcoming tactile compared with a visual target. These and similar results indicate that attention towards tactile stimuli is closely associated with modulations of both beta and alpha frequency bands in somatosensory areas (Haegens et al. 2011; Jones et al. 2010; Keil et al. 2016), as compared with neural modulations more restricted to alpha activity for visual attention (Busch et al. 2009; Samaha and Postle 2015). Together, this domain specificity in sampling frequency suggests that the underlying neural processes are likely not operating globally across sensory modalities but are rather instantiated at hierarchically lower, early sensory areas. Indeed, a number of electrophysiological studies investigating rhythmic perceptual processing as well as entrainment further support this notion by showing that behavioral fluctuations closely match oscillatory neural processes observed simultaneously in the respective sensory cortices (e.g., Landau et al. 2015; Samaha and Postle 2015; Spaak et al. 2014).

Our observed performance fluctuation speed is also in line with the related results by Baumgarten et al. (2015), who reported that the temporal resolution of somatosensory perception depends on the phase angle of 8–20 Hz oscillations just prior to stimulus presentation. They observed this effect particularly for activity in primary somatosensory areas, further supporting the notion that attentional sampling is a low‐level process located in functionally specific sensory areas (Gaillard et al. 2020).

Because our participants made responses to the target positions, the cues were also response‐congruent versus response‐incongruent, and it is therefore also possible that the performance fluctuations we found reflect rhythms in the selection of the responses corresponding to the cue locations (cf. Lingnau and Vorberg 2005). However, given that our current tactile cues were clearly supraliminal and noninformative, it is less likely that the cueing effects reflected response priming rather than attentional selection. Past studies using supraliminal but informative cues (or primes) have found that these were successfully ignored, especially with a sufficiently long inter‐stimulus interval (Ansorge et al. 2013; Kinoshita et al. 2011; see also Cheesman and Merikle 1984, for a related general argument). In contrast, rhythmic fluctuations of response priming were achieved with supraliminal primes (Huang et al. 2015; Lingnau and Vorberg 2005). Thus, it is more likely that our currently found cueing effects reflected exogenous attentional selection, as this has been demonstrated for similar stimuli in the past (e.g., Jonides 1981; Spence et al. 1998).

As we discussed above, a likely neural mechanism underlying rhythmic fluctuations in behavior in general is that the electrophysiologically observed neural oscillations in the same frequency range reflect a waxing and waning pattern of neural excitability. Notably, this interpretation does not necessarily entail the presence of discrete, “all‐or‐nothing” perceptual cycles or snapshots, but suggests a periodically changing likelihood of sensory stimuli being perceived, attended, and processed, and thus contributes to the observed fluctuations in behavior (VanRullen 2016). A similar but distinct theoretical account, most notably in the visual domain, suggests cycles of pulsed inhibition via alpha band activity, which alternate with periods of increased processing resources, particularly for salient external stimuli, associated with bursts of gamma‐band activity (Jensen et al. 2012; Landau et al. 2015).

Potentially, temporal fluctuations in hit rate and RT performance could reflect both changes in perceptual sensitivity and/or response criterion, although our current data lack the number of trials to compute these separate measures for each delay interval. However, several behavioral dense‐sampling studies have reported fluctuations specifically in response bias at both alpha band (Benedetto and Morrone 2019; Ho et al. 2017) and theta band frequency (Michel et al. 2021). Interestingly, the latter also observed fluctuations in sensitivity in the alpha range, particularly for the unattended of two locations. Likewise, Zhang et al. (2019) reported oscillations of both response bias and sensitivity in different alpha sub‐bands (10 and 8 Hz, respectively), whereas a recent MEG study (Zhou et al. 2025) mapped simultaneously occurring ongoing changes in sensitivity and response bias to alpha activity in visual and motor networks, respectively. Thus, it is possible that both processes have influenced our present findings, although strictly speaking, only sensitivity effects should be labeled as attentional.

Importantly, besides corroborating previous findings, our present data go beyond the current state of knowledge in a number of ways. First, our task required the detection of near‐threshold stimuli, as opposed to a binary discrimination between two above‐threshold stimuli as in Baumgarten et al. (2017, 2015). This suggests that attentional fluctuations not only affect how a tactile stimulus is perceived, but whether it is perceived at all. Second, we observed fluctuations not only in hit rates, but also in the RTs of correct trials. Finally, although Baumgarten et al. (2017) used a subliminal cue to reset potential ongoing fluctuations, we presently employed a clearly detectable, high‐intensity supraliminal vibration. If one considers awareness independence of an effect as a hallmark of automatic processing (cf. McCormick 1997; Mulckhuyse et al. 2007; Posner and Snyder 1975), the fact that, across past research by Baumgarten et al. (2017) and the present study, rhythmic fluctuations of performance were elicited by both subliminal and supraliminal cues, we can conclude that the fluctuations reflected a form of bottom‐up or stimulus‐driven (spatio‐temporal) selection, most likely of shifts of spatial attention. Moreover, we employed peripheral cues at the same location as the subsequent targets, which in the context of visual cueing tasks, have been consistently associated with triggering exogenous, bottom‐up attention, as opposed to central cues associated with endogenous attention (Jonides 1981; Posner 1980).

As an interesting further result, we observed overall faster RTs for correct incongruent compared with correct congruent trials. This is in line with our findings in a recent similar visual experiment (Pomper and Ansorge 2021) and might be driven by a particularly strong inhibition of return, which becomes apparent only when probing target processing at cue‐target intervals beyond those commonly investigated in cueing experiments (e.g., Cohen et al. 2005; Klein 2000; Spence et al. 2000). To investigate this further, we have additionally computed the undetrended hit rate and RT time courses because detrending likely removes the temporally slow effects of IOR. The resulting time series statistically support the presence of an inhibition of return effect (see Figure S4) and are likely the basis of the behavioral effects shown in Figure 2.

Lastly, it is worth considering why we observed significant spectral power at several frequencies between approximately 8 and 16 Hz, rather than a more condensed result in terms of a single pronounced peak. First and foremost, somatosensory oscillatory neural activity likely exhibits some interindividual variability, resulting in a range of significant frequencies across our individual participants and, consequentially, in the average spectrum. Such variability has been well documented in previous studies on somatosensory (e.g., Baumgarten et al. 2017, 2015) and visual rhythmic processing (e.g., Samaha and Postle 2015). Additionally, it is possible that our results present an overlay of more than one functional frequency band, or of a fundamental and a harmonic component, for example, at approximately 8 and 16 Hz. However, this is difficult to assess without additional electrophysiological data. Finally, the limited number of trials and the relatively low temporal resolution of the data likely also contribute to the relatively broad frequency distribution of the results.

### 4.2 Two Simultaneously Monitored Locations Are Sampled at Different Time Points

In addition to significant spectral power in performance fluctuations at several frequencies, our data indicate sampling of spatially congruent and incongruent locations at different time points. In other words, increased hit‐rate at one body location (e.g., congruent with the cue) coincided with decreased hit‐rate at another location (incongruent with the cue), and vice versa. This phase difference (mean angle of 100.5°) between congruent and incongruent time courses occurred in the alpha‐frequency range (13.2 Hz) and is in line with similar previous reports from other sensory domains. For instance, in the visual modality, research has demonstrated both alternate sampling of two simultaneously attended locations (Landau and Fries 2012) and objects (Fiebelkorn et al. 2013) in the theta frequency range. Landau et al. (2015) have shown that this alternate sampling is accompanied by parallel modulations in gamma band activity in visual areas, with cyclic power increases contralateral to the currently attended location and simultaneous power decreases contralateral to the currently unattended location. Likewise, Ho et al. (2017) have demonstrated an alternating sampling of the left and right ears, indicated by 6–8 Hz fluctuations of both perceptual sensitivity and response bias being out of phase between the two ears.

Why is our presently observed frequency, at which that phase difference between the two locations occurs, in the higher ~13 Hz range, compared with the theta range reported in the visual and auditory domains? One likely reason is that the dominant, attention‐related oscillatory activity in the visual and auditory domains is in the alpha range (8–14 Hz; Foxe and Snyder 2011; Fu et al. 2001; Pomper et al. 2015; Pomper and Chait 2017), while it is in the higher beta range (15–30 Hz) for the somatosensory domain (Bauer et al. 2012; Pomper et al. 2015; van Ede et al. 2010). Thus, if attention is required to monitor two locations simultaneously, the resulting alternate sampling manifests at half the speed of the local sensory oscillations, that is, at around 4–77 Hz in the visual domain (Re et al. 2019) compared with around 8–155 Hz in the somatosensory domain. In support of this interpretation, research in the visual domain has consequently observed further slowing of the attentional fluctuations if not only two, but three (Holcombe and Chen 2013) locations have to be monitored simultaneously. Functionally, this alternate sampling might be an energy‐efficient way to distribute limited attentional resources between different task‐relevant locations (VanRullen 2016). For the presently investigated case of somatosensory processing, a higher temporal frequency might also be advantageous and more effective in monitoring and protecting human bodily integrity from threatening and potentially harmful stimuli. Future studies could further investigate this aspect by probing potential fluctuations in subjective pain intensity of nociceptive stimuli, as attention towards pain has likewise been associated with cortical alpha‐ and beta‐band oscillations in somatosensory cortices (Höfle et al. 2013; Pomper et al. 2013).

For RTs, although the phase difference was consistent across participants, it was clustered towards zero, suggesting that response speed fluctuated in parallel at both locations. Although contrary to our hypothesis, this result is in line with a previous study from our lab, in which we observed simple RT fluctuations towards visual stimuli, in a similar frequency range (12–20 Hz) in phase for the left and right hands (Pomper and Ansorge 2021). Speculatively, this might indicate that the RT fluctuations originated at a later, motor‐related processing stage, as they were in parallel between the two hands both when stimuli were applied directly to the hands (as in the present study) and in the case of a response to a centrally presented visual stimulus (as in Pomper and Ansorge 2021).

Arguably, several additional factors have likely influenced our present results, apart from the task manipulations specific to our research question. For instance, previous research has shown that somatosensory detection performance at the left and right hands as well as the attentional modulation of evoked responses depends on how far stimulus positions are placed apart and whether selection is perceived to operate on a single or two separate objects (Eimer et al. 2004; Pang and Müller 2017). In our present case, hands were about shoulder‐width apart and operating the same keyboard, and placing them further apart and onto two separate response devices might increase the observed effects of cyclic attentional alternation. Additionally, although not within the scope of our present research, studies have also shown hemispheric differences in evoked responses, with stimuli presented to the left hand resulting in stronger and earlier responses compared with stimuli presented to the right hand (Schubert et al. 2008). In a right‐handed sample, as in our present study, this might be explained via higher attentional and processing demands for stimuli applied to the nondominant hand. However, due to our limited number of trials, we are not able to analyze differences in attentional fluctuations separately for the left and right hands.

A limitation of our experimental design is that we only tested target detection at two locations, essentially only allowing the conclusion that performance at the cued location differs from another, uncued location. Thus, it is unclear whether the observed rhythmicity in incongruent trials is specific to that location, or simply a reflection of generic attentional cycling. In other words, whether the performance patterns at the uncued hand are specific to the presently tested, task‐relevant location or similar at other uncued and unattended parts of the body remains to be investigated.

Finally, the power of the ongoing pre‐cue as well as pre‐target somatosensory alpha activity likely determines the extent to which cue and target, respectively, will get processed. Specifically, several previous studies have shown either a linear negative (Nikouline et al. 2000) or an inverted u‐shaped relationship, with optimal processing following a medium level of alpha activity (Anderson and Ding 2011; Forschack et al. 2017; Zhang and Ding 2010). Consequently, the depth of cue processing might influence the strength of the attentional reset and the presumed subsequent behavioral fluctuations, while target‐processing depth will shift the overall baseline detection level and, thus, the relevance of the preceding attentional fluctuations for task performance. In other words, very low/high prestimulus alpha might render target detection to be close to the floor/ceiling level, respectively, regardless of the current phase of attentional fluctuations.

## 5 Conclusions

Using a behavioral dense sampling approach, our present study provides strong evidence for cyclic fluctuations in somatosensory attention. The underlying speed of around 8–166 Hz fits with neural oscillations in the alpha and low beta band, which have been well documented in primary somatosensory brain areas. Crucially, we add to the existing knowledge by (1) probing the simple detection of a near threshold stimulus, (2) observing fluctuations not only in hit rates but also in RTs, (3) promoting the use of stimulus‐driven processing, most likely of attention, via a suprathreshold cue, and (4) providing evidence for the cyclic attentional distribution between two spatial locations, in line with similar findings in the visual and auditory domain.

Overall, together with recent work that identified markers of rhythmic information processing also beyond sensory perception, for example, in visual working memory (Pomper and Ansorge 2021; Schmid et al. 2022) and motor response initiation (Hogendoorn 2016; Pomper and Ansorge 2021), our study indicates that this process might be a general mechanism to deal with limited resources in the brain.

## Author Contributions

BB: formal analysis, writing – original draft; UA: conceptualization, funding acquisition, supervision, writing – review and editing; UP: conceptualization, formal analysis, funding acquisition, writing – original draft.

## Conflicts of Interest

The authors declare no conflicts of interest.

## Peer Review

The peer review history for this article is available at https://www.webofscience.com/api/gateway/wos/peer‐review/10.1111/ejn.70247.

## Supporting information


**Figure S1:** Undetrended and unsmoothed performance time‐courses from one exemplary subject (see Figure 3 for the smoothed and detrended version). **a)** The performance time courses show hit‐rates (pink trace) and response times (RTs; green trace) as a function of the variable inter‐stimulus interval (ISI), pooled across all trials. **b)** The hit‐rate time course for the congruent (pink trace) and incongruent (green trace) conditions as a function of the variable inter‐stimulus interval (ISI). **c)** Same as b), for RTs.
**Figure S2:** Performance time‐courses for 30 subjects (see main Figure 3 for the one remaining subject). For each participant, the top row shows show hit‐rates (pink trace) and response times (RTs; green trace) as a function of the variable inter‐stimulus interval (ISI), pooled across all trials. The middle row shows hit‐rate time courses for the congruent (pink trace) and incongruent (green trace) conditions as a function of the variable inter‐stimulus interval (ISI). **c)** Same as b), for RTs.
**Figure S3:** Overlay of the individual participant's spectra (black lines) as well as the mean (solid red/green line) and the permutation statistic threshold (dashed red/green line), separately for the pooled data (left column) the hit‐rate (middle column) and the response‐time data (right column).
**Figure S4:** Undetrended performance time‐courses for hit‐rates (left) and response times (right), separately for the congruent (red) and incongruent (green) conditions. The yellow line at the bottom indicates time‐windows with significant differences between the conditions. Note:To further investigate inhibition of return (IOR) as a potential cause of the overall facilitated performance in incongruent trials, we computed the undetrended hit‐rate and RT time‐courses, because detrending likely removes the temporally slow effects of IOR. Consequently, the resulting time‐series (see Figure S4 below) shows the IOR effect particularly for the hit‐rates, but to a lesser degree also for RTs, with facilitated performance first for the incongruent condition up to ~400 ms and subsequently for the congruent condition from ~750 to 1000 ms. To statistically test for this potential IOR, we performed a running t‐test between the congruent and incongruent condition for every time point. The yellow horizontal line in Figure S4 indicates datapoints with significant performance differences between the congruent (red) and the incongruent (green) condition, and supports the presence of an IOR effect in the undetrended data (which are the basis of the overall behavioral effects shown in Figure 2 of the manuscript).

## Data Availability

The data that support the findings of this study are available under https://osf.io/gpcmq/files/osfstorage.
