# Supplementary material for: Rhythmic Fluctuations in Tactile Attention
Source: Eur J Neurosci. 2025 Sep 14;62(5):e70247. doi: 10.1111/ejn.70247 (PMC12433832; doi:10.1111/ejn.70247)

Supplementary data

‚Rhythmic fluctuations in tactile attention’

(Burcu Bayram, Ulrich Ansorge, Ulrich Pomper)

**Figure S1.** Undetrended and unsmoothed performance time-courses from one exemplary subject (see Figure 3 for the smoothed and detrended version). **a)** The performance time courses show hit-rates (pink trace) and response times (RTs; green trace) as a function of the variable inter-stimulus interval (ISI), pooled across all trials. **b)** The hit-rate time course for the congruent (pink trace) and incongruent (green trace) conditions as a function of the variable inter-stimulus interval (ISI). **c)** Same as b), for RTs.


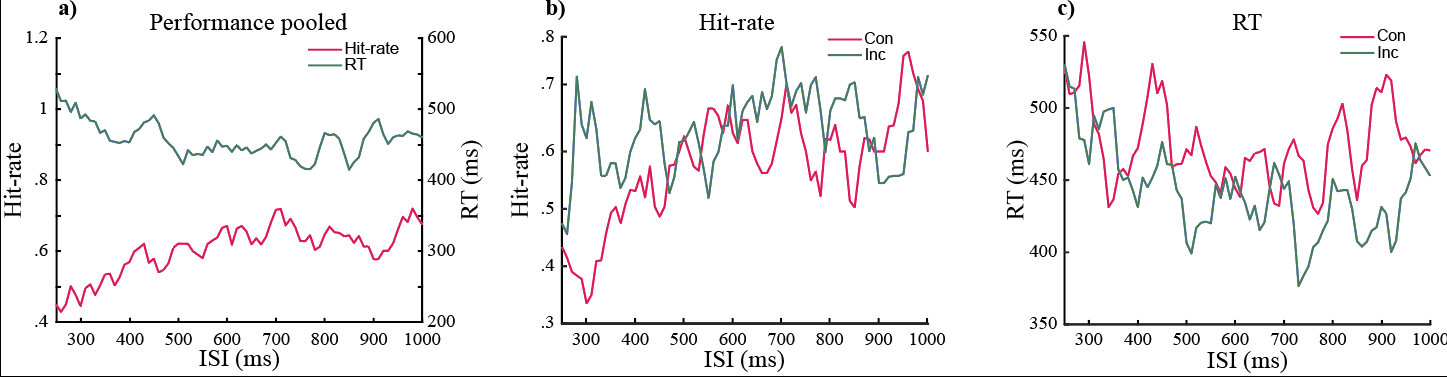


**Figure S2.** Performance time-courses for 30 subjects (see main Figure 3 for the one remaining subject). For each participant, the top row shows show hit-rates (pink trace) and response times (RTs; green trace) as a function of the variable inter-stimulus interval (ISI), pooled across all trials. The middle row shows hit-rate time courses for the congruent (pink trace) and incongruent (green trace) conditions as a function of the variable inter-stimulus interval (ISI). **c)** Same as b), for RTs.


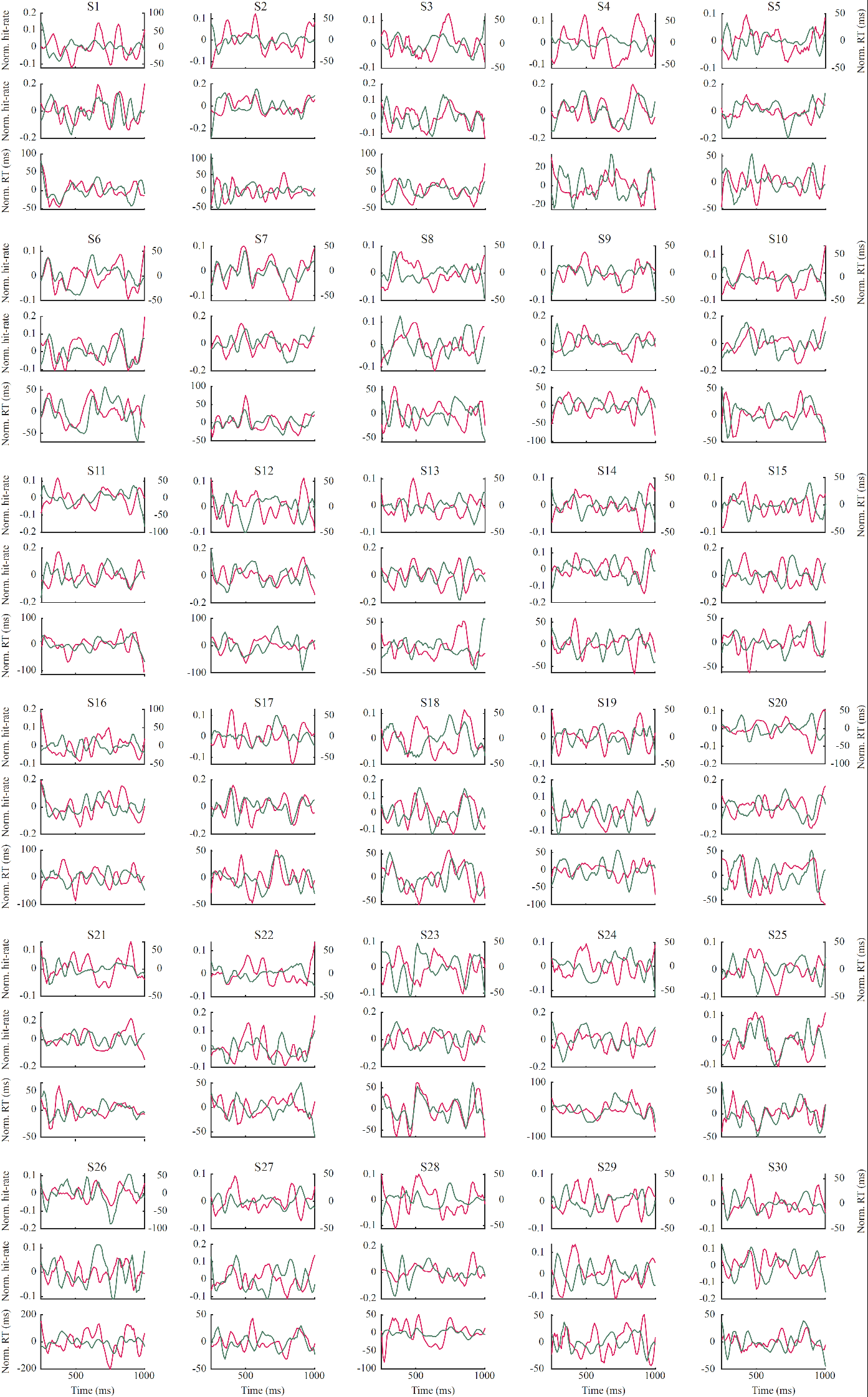


**Figure S3.** Overlay of the individual participant’s spectra (black lines) as well as the mean (solid red/ green line) and the permutation statistic threshold (dashed red/ green line), separately for the pooled data (left column) the hit-rate (middle column) and the response-time data (right column).


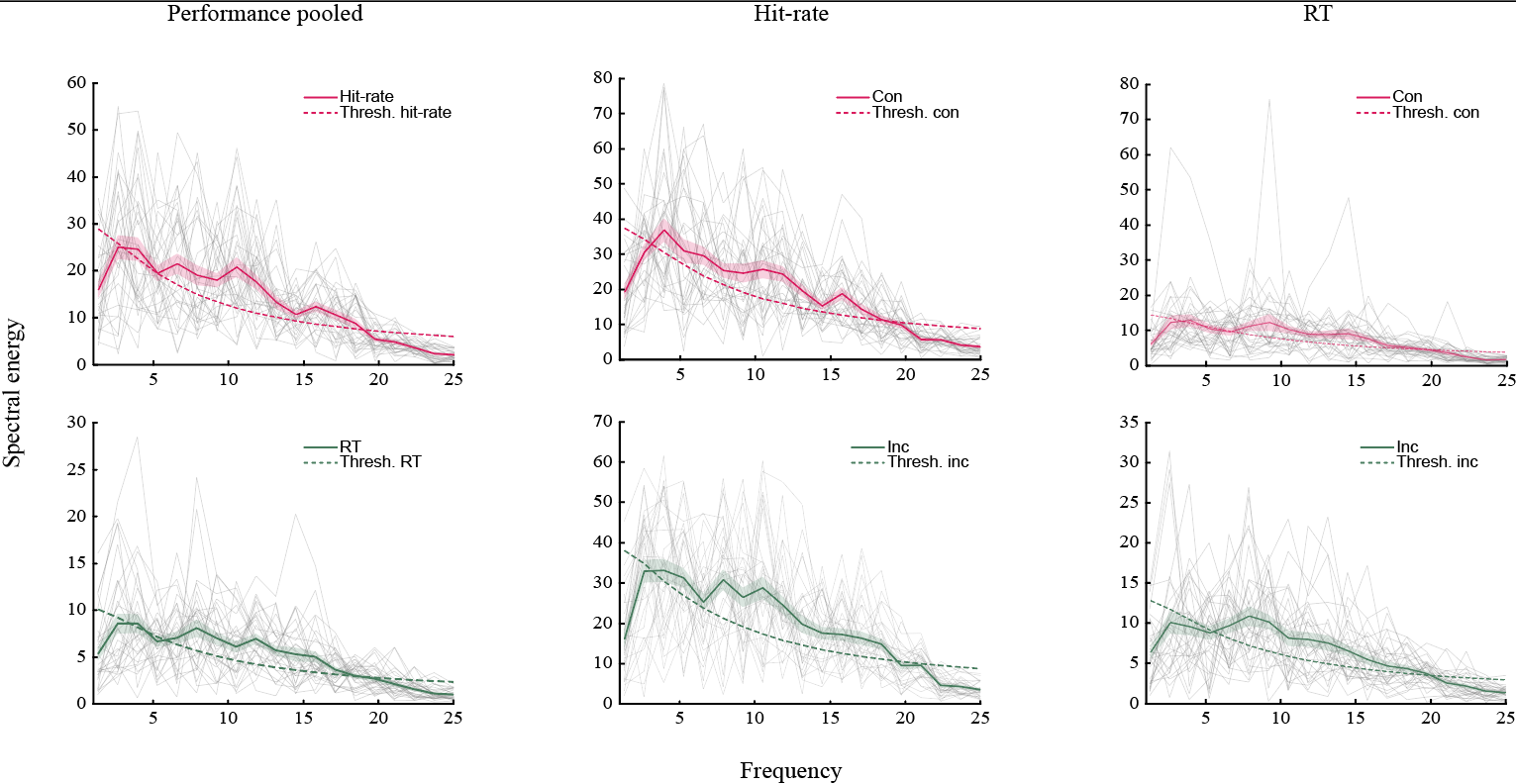


**Figure S4.** Undetrended performance time-courses for hit-rates (left) and response times (right), separately for the congruent (red) and incongruent (green) conditions. The yellow line at the bottom indicates time-windows with significant differences between the conditions.

Note: To further investigate inhibition of return (IOR) as a potential cause of the overall facilitated performance in incongruent trials, we computed the undetrended hit-rate and RT time-courses, because detrending likely removes the temporally slow effects of IOR. Consequently, the resulting time-series (see Figure S4 below) shows the IOR effect particularly for the hit-rates, but to a lesser degree also for RTs, with facilitated performance first for the incongruent condition up to ~400ms and subsequently for the congruent condition from ~750 to 1,000 ms. To statistically test for this potential IOR, we performed a running t-test between the congruent and incongruent condition for every timepoint. The yellow horizontal line in Figure S4 indicates datapoints with significant performance differences between the congruent (red) and the incongruent (green) condition, and supports the presence of an IOR effect in the undetrended data (which are the basis of the overall behavioral effects shown in Figure 2 of the manuscript).


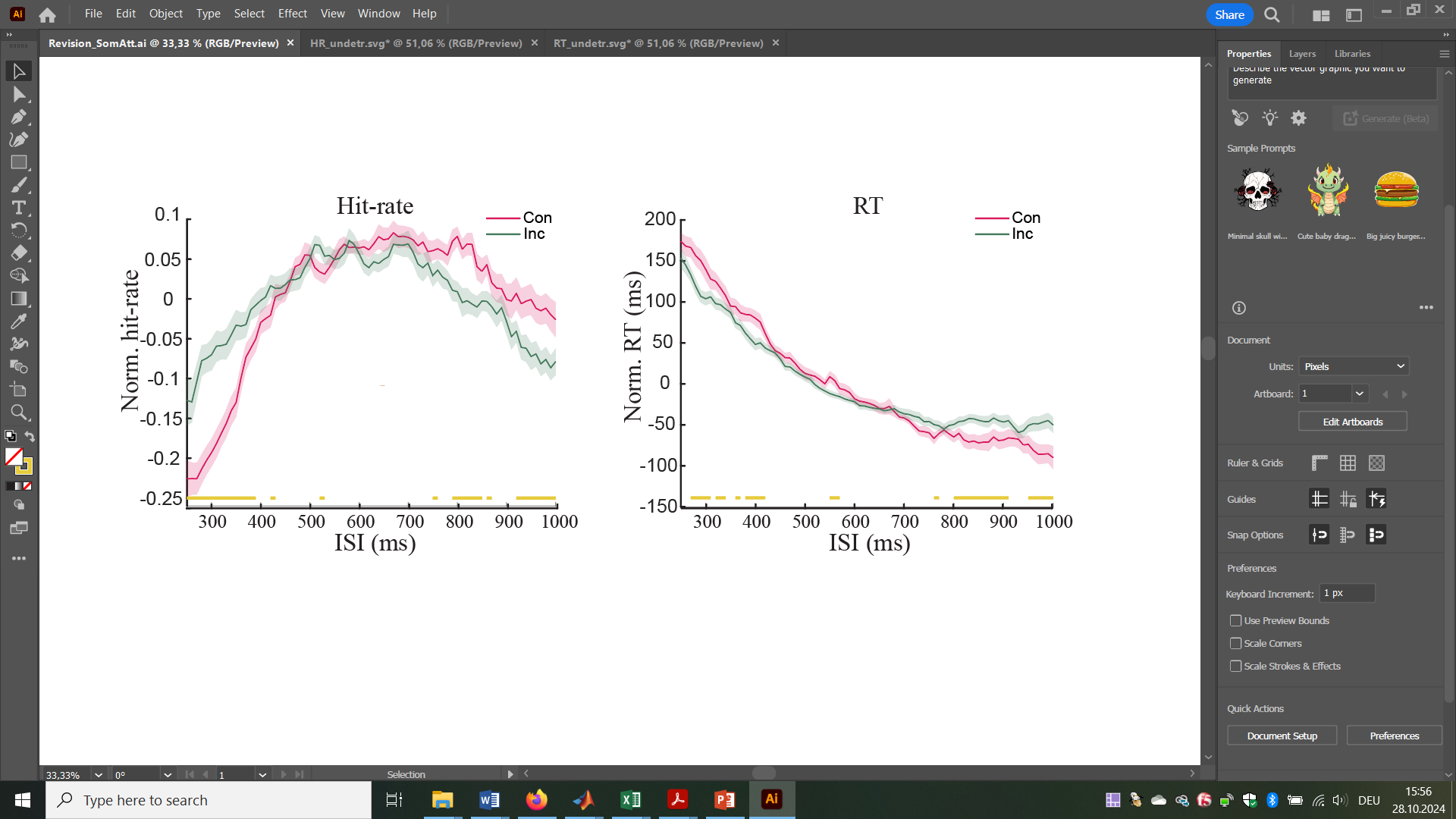

Supplement: Supplementary file 1 — Figure S1: Undetrended and unsmoothed performance time‐courses from one exemplary subject (see Figure 3 for the smoothed and detrended version). a) The performance time courses show hit‐rates (pink trace) and response times (RTs; green trace) as a function of the variable inter‐stimulus interval (ISI), pooled across all trials. b) The hit‐rate time course for the congruent (pink trace) and incongruent (green trace) conditions as a function of the variable inter‐stimulus interval (ISI). c) Same as b), for RTs. Figure S2: Performance time‐courses for 30 subjects (see main Figure 3 for the one remaining subject). For each participant, the top row shows show hit‐rates (pink trace) and response times (RTs; green trace) as a function of the variable inter‐stimulus interval (ISI), pooled across all trials. The middle row shows hit‐rate time courses for the congruent (pink trace) and incongruent (green trace) conditions as a function of the variable inter‐stimulus interval (ISI). c) Same as b), for RTs. Figure S3: Overlay of the individual participant's spectra (black lines) as well as the mean (solid red/green line) and the permutation statistic threshold (dashed red/green line), separately for the pooled data (left column) the hit‐rate (middle column) and the response‐time data (right column). Figure S4: Undetrended performance time‐courses for hit‐rates (left) and response times (right), separately for the congruent (red) and incongruent (green) conditions. The yellow line at the bottom indicates time‐windows with significant differences between the conditions. Note:To further investigate inhibition of return (IOR) as a potential cause of the overall facilitated performance in incongruent trials, we computed the undetrended hit‐rate and RT time‐courses, because detrending likely removes the temporally slow effects of IOR. Consequently, the resulting time‐series (see Figure S4 below) shows the IOR effect particularly for the hit‐rates, but to a lesser degree also for RTs, [file EJN-62-0-s001.docx]
